# Meropenem-Vaborbactam Resistance Selection, Resistance Prevention, and Molecular Mechanisms in Mutants of KPC-Producing Klebsiella pneumoniae

**DOI:** 10.1128/AAC.01694-17

**Published:** 2017-11-22

**Authors:** Dongxu Sun, Debora Rubio-Aparicio, Kirk Nelson, Michael N. Dudley, Olga Lomovskaya

**Affiliations:** The Medicines Company, San Diego, California, USA

**Keywords:** vaborbactam, Klebsiella pneumoniae carbapenemases, KPC, resistance development, single-step mutants

## Abstract

Vaborbactam (formerly RPX7009) is a new β-lactamase inhibitor based on a cyclic boronic acid pharmacophore with potent inhibitory activity against Klebsiella pneumoniae carbapenemases (KPC). It has been developed in combination with meropenem. The objective of these studies was to identify the concentrations of both agents associated with the selection or prevention of single-step mutations leading to reduced sensitivity to the combination and to characterize the selected mutations. Eighteen strains of KPC-producing Klebsiella pneumoniae with various degrees of sensitivity to meropenem (MICs, 8 to 512 μg/ml) and meropenem-vaborbactam (MICs, ≤0.06 to 32 μg/ml) and preexisting resistance mechanisms were selected from a worldwide collection of isolates recovered from surveillance studies, emphasizing strains for which MICs were in the upper range of the meropenem-vaborbactam MIC distribution. Meropenem and vaborbactam at 8 μg/ml each suppressed the drug resistance mutation frequency to <1 × 10^−8^ in 77.8% (14/18) of strains, and all strains were inhibited when the meropenem concentration was increased to 16 μg/ml. Mutants selected at lower drug concentrations showed phenotypes associated with previously described carbapenem resistance mechanisms, including *ompK36* inactivation in mutants selected from OmpK36-proficient strains and an increased *bla*_KPC_ gene copy number in strains with partially functional *ompK36*. No mutations in the coding region of *bla*_KPC_ were identified. These data indicate that the selection of mutants with reduced sensitivity to meropenem-vaborbactam from KPC-producing Klebsiella pneumoniae strains is associated with previously described mechanisms involving porin mutations and the increase in the *bla*_KPC_ gene copy number and not changes in the KPC enzyme and can be prevented by the drug concentrations achieved with optimal dosing of the combination.

## INTRODUCTION

Carbapenems are β-lactam antibiotics often used in the treatment of serious Gram-negative bacterial infections, particularly those caused by isolates that produce extended-spectrum β-lactamases (ESBLs) and that are resistant to other β-lactam antibiotics. Meropenem is an injectable carbapenem with excellent stability to ESBLs and good safety and tolerability that has been used around the world since 1994 (1, 2). The widespread emergence of carbapenemases and resistance to carbapenems worldwide threatens their clinical use (3–6). Enterobacteriaceae that produce Klebsiella pneumoniae carbapenemases (KPC) are the leading cause of carbapenem-resistant infections in the United States and other regions, creating an urgent public health threat that requires immediate action (6–10).

Vaborbactam (formerly called RPX7009) is a new inhibitor of class A and class C β-lactamases based on a cyclic boronic acid pharmacophore with particularly potent activity against KPC (11). Several studies have demonstrated that vaborbactam enhances the *in vitro* potency of carbapenem antibiotics against KPC-producing Enterobacteriaceae (11–15). The excellent safety and tolerability of vaborbactam were demonstrated in a phase 1 clinical trial: no serious adverse effects were observed at doses up to 2,000 mg (16). Vaborbactam in combination with meropenem was recently approved by the Food and Drug Administration for the treatment of complicated urinary tract infections, including pyelonephritis (17, 18).

The evaluation of drug resistance mechanisms that contribute to decreased sensitivity and the identification of antibiotic concentrations that reduce the selection of drug-resistant mutants are important steps in the selection of a dosing regimen for any new agent. This also applies to β-lactam–β-lactamase inhibitor combinations, which are more complex, given that drug resistance mechanisms may affect the β-lactamase inhibitor, the partner β-lactam, or both. In this study, we evaluated the frequency of selection of single-step meropenem-vaborbactam (M-V)-resistant mutants in KPC-producing strains of K. pneumoniae, determined the concentrations that reduced the selection frequency, and characterized the molecular mechanisms associated with increased meropenem-vaborbactam MICs. The strains selected for study were phylogenetically diverse and included those with preexisting resistance mechanisms whose genotypes and phenotypes would be expected to present a high risk of resistance development.

## RESULTS

### Characterization of strains used in resistance development studies.

Table 1 describes the 18 KPC-producing K. pneumoniae strains used in this study. They were isolated during large-scale surveillance studies and were selected for further study as they represented strains with a broad distribution of M-V MICs, ranging from ≤0.06 to 32 μg/ml, including nine strains with MICs of ≥2 μg/ml. Two strains in the latter group had M-V MICs of 16 and 32 μg/ml, respectively. On the basis of the results of surveillance studies, strains with M-V MICs of ≥16 μg/ml represent ≤0.5% of KPC-producing Enterobacteriaceae (14, 15). The collection was phylogenetically diverse, as it included strains from several multilocus sequence types (MLSTs); it also contained several strains from the internationally spread sequence type 258 (ST-258) lineage (19) (Table 1). The collection was equally divided between KPC-2 and KPC-3 variants; all strains carried at least 2 other β-lactamase genes, with narrow-spectrum TEM-1 and SHV-11 variants commonly being present among the isolates. Several strains contained class A extended-spectrum β-lactamases, such as CTX-M-14/CTX-M-15 and SHV-12, or the class C β-lactamase CMY-2. Twelve strains had tigecycline MICs of ≤1 μg/ml, suggesting a basal level of activity of the multidrug resistance efflux pump AcrAB-TolC (20).

**TABLE 1:** K. pneumoniae strains used in this study

Strain	Strain isolation		MLST^*a*^	β-Lactamase genes	Major porin mutation^*b*^		MIC (μg/ml)		
	Yr	Country			*ompK35*	*ompK36*	Tigecycline	Meropenem	
								Alone	With VAB^*c*^ at 8 μg/ml
KPM1275	This study	This study	ST-493	KPC-3, TEM-1, SHV-24	FL	FL	0.5	16	≤0.06
KP1004	2004	USA	ST-258	KPC-2, TEM-1, SHV-11	FS_aa42	FL	1	32	≤0.06
KP1008	2004	USA	ST-14	KPC-2, TEM-1, SHV	FL	FL	0.5	4	≤0.06
KP1083	2007	USA	ST-2020	KPC-3, SHV-1, TEM-1	No PCR product	FL	0.5	16	≤0.06
KP1088	2010	USA	ST-258	KPC-3, SHV-1, TEM-1	FS_aa42	FL	1	16	≤0.06
KP1087	2010	USA	ST-307	KPC-2, CTX-M-15, SHV-11, TEM-1	FS_aa208	GD	0.5	16	0.125
KP1074	2008	Israel	ST-512	KPC-3 TEM-1, SHV-11	FS_aa42	GD	0.5	128	0.5
KP1084	2007	USA	ST-258	KPC-3, TEM-1, SHV-11	FS_aa42	GD	0.5	64	0.5
KP1093	2009	UK	ST-258	KPC-3 TEM-1, SHV-11	FS_aa42	GD	1	>128	0.5
KP1196	2013	USA	ST-307	KPC-2, TEM-1, SHV, CTX-M-15, CMY-2	FS_aa208	GD	2	128	1
KP1099	2011	China	ST-11	KPC-2, CTX-M-14, SHV-11, SHV-12	FS_aa29	GD	1	512	2
KP1193	2013	USA	ST-12	KPC-3, TEM, SHV	FL	TAA_aa131	2	512	2
KP1200	2012	USA	ST-258	KPC-3, TEM, SHV	FS_aa42	FS_aa28	2	512	2
KP1094	2010	UK	ST-498	KPC-2, TEM-1, LEN-17	TAG_aa230	TAG_aa92	0.5	512	4
KP1100	2011	Italy	ST-512	KPC-3, TEM-1, SHV-11	FS_aa42	GD	2	512	4
KP1194	2013	USA	ST-258	KPC-2, TEM, SHV	FS_aa42	IS*5* promoter	2	512	8
KP1096	2011	UK	ST-1162	KPC-2, TEM-181, SHV-11	L63V, E132K	IS102_nt126	2	512	16
KP1092	2010	USA	ST-258	KPC-2, TEM-1, SHV-11, SHV-12	FS_aa42	IS*5* promoter	0.5	>512	32

a MLST, multilocus sequence type.

b Major porin sequence: FL, full length; FS_aaXX, frameshift at the amino acid number indicated by XX; TAA_aaXX and TAG_aaXX, nonsense mutations at the amino acids indicated by XX; GD, glycine-aspartic acid duplication at positions 136 and 137; L63V, leucine-to-valine substitution at amino acid 63; E132K, glutamic acid-to-lysine substitution at amino acid 132; IS_nt126, insertion sequence at nucleotide 126; IS*5* promoter, insertion of insertion sequence 5 in the promoter region.

c VAB, vaborbactam.

Porin gene sequence analyses showed various combinations of mutations in the major porin genes *ompK35* and *ompK36* (Table 1). Five strains with M-V MIC values of ≤0.06 μg/ml had full-length *ompK36* genes and either full-length or nonfunctional *ompK35* genes. Five strains with M-V MICs of between 0.125 and 1 μg/ml had a nonfunctional OmpK35 porin due to frameshift mutations at amino acid 42 or 208 and a partially functional OmpK36 porin containing a glycine (G) and aspartic acid (D) amino acid duplication (GD duplication) at positions 136 and 137. Of eight strains with higher M-V MICs, three (strains KP1200, KP1094, and KP1096) contained double loss-of-function porin mutations with various frameshift mutations, nonsense mutations, nonsynonymous substitutions, deletions, or insertions. Strains KP1194 and KP1092, for which the M-V MICs were 8 μg/ml and 32 μg/ml, respectively, had a nonfunctional OmpK35 and an IS*5* insertion element in the *ompK36* gene promoter region, which is known to drastically reduce *ompK36* expression (21). KP1099 (M-V MIC, 2 μg/ml) and KP1100 (M-V MIC, 4 μg/ml) had a nonfunctional OmpK35 and OmpK36 with a GD duplication. Strain KP1193 (M-V MIC, 2 μg/ml) had a full-length OmpK35 but carried a nonsense mutation in the gene *ramR* that may lead to OmpK35 downregulation (22).

### Frequencies of single-step mutants with increased meropenem-vaborbactam MICs and conditions that reduce selection of resistant mutants.

Table 2 summarizes the frequencies of emergence of mutants selected from the 18 parental strains using combinations of meropenem and vaborbactam at various concentrations. A frequency of <1 × 10^−8^ was used as a threshold for reduced mutant selection. Two strains, KPM1275 and KP1008, with M-V MICs of ≤0.06 μg/ml required the lowest concentration of meropenem and vaborbactam (2 μg/ml each) to suppress the selection of drug-resistant mutants. Meropenem at 4 μg/ml combined with vaborbactam at 8 μg/ml was required to reduce resistance selection in five strains with M-V MIC values ranging from ≤0.06 μg/ml to 0.5 μg/ml. Meropenem and vaborbactam at 8 μg/ml each reduced the mutant emergence frequency to <1 × 10^−8^ in seven more strains with M-V MICs ranging from 0.5 μg/ml (KP1074) to 8 μg/ml (KP1194). KP1099 and KP1100 (M-V MIC values, 2 μg/ml and 4 μg/ml, respectively) required 16 μg/ml of meropenem combined with 8 μg/ml of vaborbactam or 8 μg/ml of meropenem combined with 16 μg/ml of vaborbactam to reduce the mutant emergence frequency to below 1 × 10^−8^. KP1096 and KP1092, the most resistant strains included in this study, with M-V MICs of 16 μg/ml and 32 μg/ml, respectively, required meropenem at 16 μg/ml combined with at least 8 μg/ml of vaborbactam to reduce resistance selection to a frequency of <1× 10^−8^.

**TABLE 2:** Mutant emergence frequency in KPC-producing K. pneumoniae strains exposed to various concentrations of meropenem and vaborbactam^*a*^

Strain	MER MIC (μg/ml)		MER concn/VAB concn (μg/ml) used for selection^*b*^	Mutant emergence frequency^*b*^
	Alone	With VAB at 8 μg/ml		
KPM1275	16	≤0.06	2/0.5	2.2 × 10^−7^
			0.5/2	2.0 × 10^−8^
			**2/2**	**<1 × 10^−8^**
			**4/2**	**<1 × 10^−8^**
			**8/2**	**<1 × 10^−8^**
			**2/4**	**<1 × 10^−8^**
			**4/4**	**<1 × 10^−8^**
			**8/4**	**<1 × 10^−8^**
			**2/8**	**<1 × 10^−8^**
			**4/8**	**<1 × 10^−8^**
			**8/8**	**<2 × 10^−9^**
KP1008	4	≤0.06	2/0.5	8.4 × 10^−7^
			0.5/2	7.3 × 10^−7^
			**2/2**	**<1** × **10^−8^**
			**4/2**	**<1** × **10^−8^**
			**8/2**	**<1** × **10^−8^**
			**2/4**	**<1 × 10^−8^**
			**4/4**	**<1 × 10^−8^**
			**8/4**	**<1 × 10^−8^**
			**2/8**	**<1 × 10^−8^**
			**4/8**	**<1 × 10^−8^**
			**8/8**	**<2 × 10^−9^**
KP1004	32	≤0.06	2/2	8.0 × 10^−8^
			4/2	8.0 × 10^−8^
			8/2	8.0 × 10^−8^
			2/4	4.0 × 10^−8^
			4/4	4.0 × 10^−8^
			**8/4**	**<1** × **10^−8^**
			**2/8**	**<1 × 10^−8^**
			**4/8**	**<1 × 10^−8^**
			**8/8**	**<3 × 10**^−10^
KP1083	16	≤0.06	2/2	7.4 × 10^−7^
			4/2	7.7 × 10^−7^
			8/2	4.8 × 10^−7^
			2/4	3.9 × 10^−7^
			4/4	3.0 × 10^−7^
			8/4	2.0 × 10^−8^
			2/8	4.0 × 10^−8^
			**4/8**	**<1.0 × 10^−8^**
			**8/8**	**<1.0 × 10^−9^**
KP1088	16	≤0.06	2/2	2.1 × 10^−7^
			4/2	2.2 × 10^−7^
			8/2	1.7 × 10^−7^
			2/4	1.1 × 10^−7^
			4/4	4.0 × 10^−8^
			8/4	2.0 × 10^−8^
			**2/8**	**<1.0 × 10^−8^**
			**4/8**	**<1.0 × 10^−8^**
			**8/8**	**<1.8 × 10^−9^**
KP1087	16	0.125	2/2	4.0 × 10^−6^
			4/2	5.0 × 10^−7^
			8/2	5.0 × 10^−8^
			2/4	2.0 × 10^−8^
			4/4	2.0 × 10^−8^
			**8/4**	**<1** × **10^−8^**
			2/8	2.0 × 10^−8^
			**4/8**	**<1.0** × **10^−8^**
			**8/8**	**<2.9 × 10^−9^**
KP1074	128	0.5	2/2	2.0 × 10^−5^
			4/2	6.0 × 10^−6^
			8/2	5.0 × 10^−6^
			2/4	3.3 × 10^−6^
			4/4	2.8 × 10^−6^
			8/4	5.0 × 10^−7^
			2/8	3.7 × 10^−7^
			4/8	2.0 × 10^−8^
			**8/8**	**<3.6** × **10^−9^**
KP1084	64	0.5	2/2	3.0 × 10^−5^
			4/2	2.0 × 10^−5^
			8/2	5.0 × 10^−6^
			2/4	2.0 × 10^−7^
			4/4	1.0 × 10^−7^
			8/4	5.0 × 10^−8^
			**2/8**	**<1 × 10^−8^**
			**4/8**	**<1 × 10^−8^**
			**8/8**	**<1 × 10^−9^**
KP1093	>128	0.5	2/2	5.0 × 10^−6^
			4/4	3.0 × 10^−7^
			**8/8**	**<5** × **10**^−10^
KP1196	128	1	2/2	TMC^*c*^
			4/4	6.2 × 10^−8^
			**8/8**	**<8** × **10**^−10^
KP1099	512	2	8/8	8.9 × 10^−7^
			**16/8**	**<6.7** × **10^−9^**
			**8/16**	**<6.7 × 10^−9^**
			**16/16**	**<6.7 × 10^−9^**
KP1193	512	2	**8/8**	**<2.6** × **10^−9^**
			**16/8**	**<2.6** × **10^−9^**
			**8/16**	**<2.6** × **10^−9^**
			**16/16**	**<2.6** × **10^−9^**
KP1200	512	2	**8/8**	**<2.1** × **10^−9^**
			**16/8**	**<2.1** × **10^−9^**
			**8/16**	**<2.1** × **10^−9^**
			**16/16**	**<2.1** × **10^−9^**
KP1094	512	4	**8/8**	**<1.6** × **10^−9^**
			**16/8**	**<1.6** × **10^−9^**
			**8/16**	**<1.6** × **10^−9^**
			**16/16**	**<1.6** × **10^−9^**
KP1100	512	4	8/8	4.5 × 10^−8^
			**16/8**	**<3.0** × **10^−9^**
			**8/16**	**<3.0** × **10^−9^**
			**16/16**	**<3.0** × **10^−9^**
KP1194	512	8	**8/8**	**<5.6 × 10^−9^**
			**16/8**	**<5.6** × **10^−9^**
			**8/16**	**<5.6** × **10^−9^**
			**16/16**	**<5.6** × **10^−9^**
KP1096	512	16	8/8	4.3 × 10^−6^
			**16/8**	**<2.4** × **10^−9^**
			8/16	1.7 × 10^−7^
			**16/16**	**<2.4** × **10^−9^**
KP1092	>512	32	8/8	2.3 × 10^−6^
			**16/8**	**<2.3** × **10^−9^**
			8/16	1.4 × 10^−7^
			**16/16**	**<2.3** × **10^−9^**

a MER, meropenem; VAB, vaborbactam.

b Results in bold represent the concentrations and frequencies with a resistance frequency of <1 × 10^−8^.

c TMC, too many to count.

### Meropenem-vaborbactam MICs for selected mutants.

Table 3 lists the meropenem MICs for mutants that were selected for further characterization and that were exposed to vaborbactam at various concentrations. In general, mutants isolated from the parental strains with functional copies of both the OmpK35 and OmpK36 porins (strains KPM1275 and KP1008) appeared to be less resistant to meropenem-vaborbactam than those isolated from parents with preexisting mutations. This is consistent with the fact that the concentrations of meropenem and vaborbactam required to prevent mutant emergence from those strains with fully functional porins were the lowest. The M-V MIC values for the mutants selected from KPM1275 and KP1008 ranged from 0.25 μg/ml to 1 μg/ml, representing 4- to 16-fold increases in the M-V MICs compared to those for the parental strains. In contrast, the M-V MICs recorded for mutants selected from strains with a nonfunctional OmpK35 and a fully functional OmpK36 (strains KP1004, KP1083, and KP1088) ranged from 1 μg/ml to 8 μg/ml, corresponding to 16- to 128-fold increases in the M-V MIC values between the mutant and parental strains.

**TABLE 3:** Evaluation of meropenem-vaborbactam susceptibility and genetic changes associated with increased meropenem-vaborbactam MICs in K. pneumoniae laboratory mutants

Parent and mutant	Meropenem MIC (μg/ml) in presence of VAB (μg/ml) at:					Porin gene mutation(s)^*a*^		*bla*_KPC_ copy no.^*b*^
	Alone	2	4	8	16	*ompK35*	*ompK36*	
KPM1275	32	≤0.06	≤0.06	≤0.06	≤0.06	Same as ATCC 43816 sequence	Same as ATCC 43816 sequence	
KPM1852	>64	2	0.5	0.25	0.125	Same as ATCC 43816 sequence	Same as ATCC 43816 sequence	
KPM1853	>64	16	4	1	1	Same as ATCC 43816 sequence	FS_aa324	
KP1008	4	≤0.06	≤0.06	≤0.06	≤0.06	Same as ATCC 43816 sequence	Same as ATCC 43816 sequence	
KPM1837	64	4	1	0.5	0.25	Same as ATCC 43816 sequence	No PCR product	
KPM1838	128	4	2	0.5	0.5	Same as ATCC 43816 sequence	TAG_aa298	
KPM1839	512	2	4	0.5	0.5	Same as ATCC 43816 sequence	V317D	
KP1008-12	>64	8	ND	0.5	ND	Same as ATCC 43816 sequence	FS_aa55	
KP1004	32	0.125	≤0.06	≤0.06	≤0.06	FS_aa42	Full length	
KPM1835	>64	256	32	4	1	Not done	FS_aa2	
KPM1836	>64	128	16	2	1	Not done	No PCR product	
KP1004-11	>64	>64	ND	4	ND	Not done	FS_aa45	
KP1004-13	>64	>64	ND	4	ND	Not done	TAG_aa70	
KP1074	64	4	1	0.25	0.125	FS_aa42	GD duplication	1.0
KPM1840	512	128	32	4	1	Not done	GD duplication	8.4
KPM1841	128	4	2	0.5	1	Not done	GD duplication	1.9
KPM2145	256	128	32	1	0.125	Not done	GD duplication	3.3
KPM2146	256	128	32	1	0.5	Not done	GD duplication	2.3
KPM2163	256	128	128	8	0.25	Not done	GD duplication	3.7
KPM2310	>64	16	2	1	0.5	Not done	GD duplication	4.6
KP1083	32	1	0.125	≤0.06	≤0.06	IS_nt174	Full length	
KPM1842	512	128	8	1	0.5	Not done	FS_aa161	
KPM1843	512	128	8	1	0.5	Not done	No PCR product	
KPM1844	512	128	8	1	0.5	Not done	FS_aa213	
KP1088	16	≤0.06	≤0.06	≤0.06	≤0.06	FS_aa42	Full length	
KPM1849	>64	128	4	1	≤0.5	Not done	TAG_aa227	
KP1088-11	>64	>64	ND	4	ND	Not done	TAG_ aa170	
KP1088-12	>64	>64	ND	8	ND	Not done	L361Q	
KP1084	64	16	1	0.25	0.125	FS_ aa42	GD duplication	1.0
KPM1845	256	32	8	0.5	0.5	Not done	GD duplication	6.3
KPM1846	256	64	16	2	1	Not done	GD duplication	9.7
KPM1847	256	64	16	1	1	Not done	GD duplication	4.9
KPM2164	128	128	64	2	0.25	Not done	GD duplication	1.9
KPM2313	>64	32	4	2	1	Not done	GD duplication	5.2
KPM2314	>64	16	4	2	0.5	Not done	GD duplication	5.4
KP1087	32	4	1	0.25	0.125	FS_aa208	GD duplication	1.0
KP1848	>64	32	4	1	0.5	Not done	GD duplication	0.9
KP1093	>64	1	0.5	0.25	ND	FS_aa208	GD duplication	1.0
KPM2802	>64	>64	32	2	ND	Not done	GD duplication	3.4
KPM2803	>64	>64	16	1	ND	Not done	GD duplication	4.3
KPM2804	>64	>64	16	1	ND	Not done	GD duplication	1.0
KPM2805	>64	8	2	4	ND	Not done	GD duplication	0.9
KP1196	128	ND	ND	1	ND	FS_aa208	GD duplication	1.0
KPM2879	256	ND	ND	8	ND	Not done	GD duplication	2.3
KP1099	512	32	8	2	1	FS_aa29	GD duplication	1.0
KPM2893	>512	256	128	32	2	Not done	GD duplication	5.0
KPM2894	>512	256	256	64	4	Not done	GD duplication	2.1
KPM2895	>512	256	256	64	2	Not done	GD duplication	3.8
KPM2896	>512	256	256	64	4	Not done	GD duplication	7.5
KP1100	>64	16	8	4	4	FS_aa42	GD duplication	1.0
KPM2806	>64	>64	>64	32	4	Not done	GD duplication	3.1
KPM2807	>64	>64	>64	32	4	Not done	GD duplication	4.1
KPM2808	>64	>64	>64	>64	8	Not done	GD duplication	7.4
KP1096	512	256	64	16	4	L63V, E132K	IS102_nt126	1.0
KPM2329	>512	>512	512	64	16	L63V, E132K	IS102_nt126	0.8
KPM2330	>512	>512	256	64	8	L63V, E132K	IS102_nt126	1.1
KP1092	>512	512	128	32	8	FS_aa42	IS*5* at nucleotide −45 (promoter insertion)	1.0
KPM2327	>512	>512	512	64	16	FS_42	IS*5* at nucleotide −45 (promoter insertion)	1.4
KPM2328	>512	>512	512	64	16	FS_42	IS*5* at nucleotide −45 (promoter insertion)	1.1

a Mutations observed in the porin genes. ND, not determined; FS_aaXX, frameshift at the amino acid number indicated by XX; IS_nt174, 2-kb insertion sequence at nucleotide 176; TAG_aaXX, nonsense mutations at the amino acid number indicated by XX; V317D, valine-to-aspartic acid substitution at amino acid 317; FL, full-length protein; ΔVG_aa320, deletion of valine and glycine at amino acids 320 and 321; L265P, leucine-to-proline substitution at amino acid 265; Δnt163, 771-bp deletion starting at nucleotide 163; GD duplication, duplication of amino acids G (Gly) and D (Asp) at amino acids 136 and 137; L63V, leucine-to-valine substitution at amino acid 63; E132K, glutamic acid-to-lysine substitution at amino acid 132; IS102_nt126, insertion sequence at nucleotide 126; ND, not determined.

b Relative *bla*_KPC_ copy number between parents and mutants when the number for the parental strain is normalized to 1. The value was not determined for the strains with blank entries.

The M-V MICs for mutants selected from five of the seven parents with a partially functional OmpK36 due to the presence of a GD duplication (strains KP1074, KP1084, KP1087, KP1093, and KP1196; M-V MIC values, 0.125 to 1 μg/ml) ranged from 0.5 to 8 μg/ml, leading to 4- to 32-fold increases in the M-V MICs. Mutants selected from the two remaining strains, KP1099 and KP1100, had M-V MICs of 32 to 64 μg/ml when meropenem MICs were determined with vaborbactam at 8 μg/ml and 2 to 8 μg/ml when the vaborbactam concentration was doubled to 16 μg/ml (Table 3). Strains KP1096 and KP1092, the parents with the highest base M-V MICs, produced mutants with 2- to 4-fold increases in M-V MICs, representing the smallest MIC differences recorded in the study.

### Changes in major porin gene sequences.

Table 3 compares the major porin *ompK35* and *ompK36* gene sequences between the parents and mutants. No changes in the *ompK35* genes of mutants selected from the two K. pneumoniae strains with wild-type genes (strains KPM1275 and KP1008) were recorded. However, the genetic background of the parental *ompK36* gene impacted the molecular changes observed in the mutants. Many mutants (12 of 16, 75%) selected from 5 parents with wild-type *ompK36* genes (strains KPM1275, KP1008, KP1004, KP1083, and KP1088) had nonfunctional OmpK36 proteins due to frameshift mutations, nonsense mutations, nonsynonymous substitutions, or deletions in the coding region. The specific mutations could not be evaluated in 18.8% (3 of 16) of the mutants due to failed DNA amplification reactions, potentially due to large insertions. In contrast, no additional changes were recorded in the *ompK36* genes of the mutants selected from parental strains containing the *ompK36* gene variant that encodes the partially functional OmpK36 porin containing a GD duplication (strains KP1074, KP1084, KP1087, KP1093, KP1196, KP1099, and KP1100). No additional changes in the *ompK36* gene sequences were also seen in mutants selected from KP1092, which had decreased *ompK36* expression, and KP1096, in which this gene was inactivated by the insertion of IS*102* at nucleotide 126 of the *ompK36* coding region.

### *bla*_KPC_ gene copy number.

We investigated possible *bla*_KPC_ coding sequence, promoter, and gene copy number changes for the mutants whose M-V MIC increases could not be linked to mutations in the *ompK35* and *ompK36* genes. Sequencing of the *bla*_KPC_ gene promoter and coding regions revealed no differences between the parental and mutant strains. We measured the *bla*_KPC_ gene copy number in mutants selected from parents with GD duplication-containing OmpK36 porins and found that 88% (22 of 25) showed an approximately 2- to 10-fold increase in *bla*_KPC_ gene copy number (Table 3). No *bla*_KPC_ gene copy number increases were found in mutants selected from the two most resistant strains, KP1092 and KP1096.

### Changes in KPC-containing plasmids.

**FIG 1:**
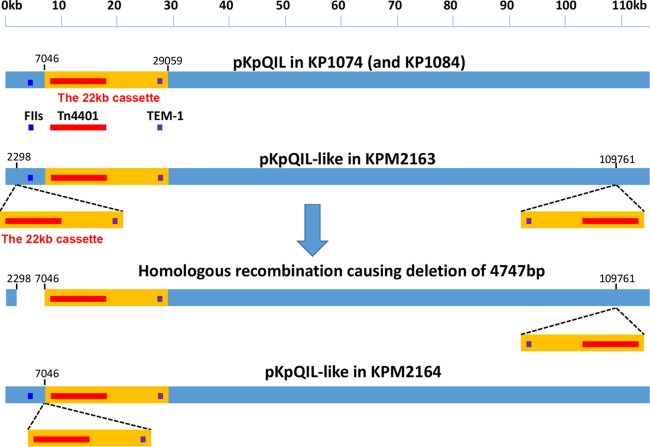
Rearrangement of the pKpQIL-like plasmid in strains KPM2163 and KPM2164. In KPM2163, an approximately 22-kb region of pKpQIL (nucleotides 7046 to 29059) was duplicated and inserted at nucleotide 2298 and nucleotide 109761. The plasmid had 3 copies of Tn*4401*. In some cells, the 22-kb copy at nucleotide 2298 and the original 22-kb copy underwent homologous recombination, resulting in the looping out of a 22-kb copy and the 4,747-bp sequence (from nucleotide 2298 to nucleotide 7046) between the 2 copies. The resulting plasmid had 2 copies of Tn*4401*. In KPM2164, the same 22-kb cassette was duplicated *in situ* to produce a tandem direct repeat of the 22 kb. The resulting plasmid had 2 copies of Tn*4401*.

Eight mutants derived from parental strains KP1074, KP1084, and KP1099 were selected to explore the genetic events that might have increased the *bla*_KPC_ gene copy number. Table 4 and Fig. 1 summarize the three molecular changes identified in the mutants.

**TABLE 4:** Changes in pKpQIL KPC-carrying plasmid in K. pneumoniae mutants with increased *bla*_KPC_ gene copy number

Parent and mutant	Type of molecular change	Details
KP1074		
KPM2163	Plasmid rearrangement	22 kb of pKpQIL (nucleotides 7046–29059) containing Tn*4401* duplicated at nucleotide 2298 and nucleotide 109761; recombination between the 22 kb at nucleotide 2298 and the native 22-kb fragment resulted in the deletion of a 4,748-bp fragment (nucleotides 2298 to 7048) and the generation of 3 Tn*4401* copies
KPM2310	Tn*4401* transposition to a smaller plasmid	Tn*4401* inserted into ColST258 at nucleotide 8367 (within the *mobC* gene)
KP1084		
KPM2164	Plasmid rearrangement	22 kb of pKpQIL (nucleotides 7046–29,059) containing Tn*4401* was duplicated *in situ*, resulting in the generation of tandem repeats
KPM2313	Tn*4401* transposition to a smaller plasmid	Tn*4401* inserted into ColST258 at nucleotide 3108 within the Tn*3* transposase gene; the new plasmid lost a 6,221-bp fragment
KPM2314	Tn*4401* transposition to a smaller plasmid	Tn*4401* inserted into ColST258 at nucleotide 4005 within the cloacin gene
KP1099		
KPM2893	Plasmid rearrangement	IS*5* inserted at nucleotide 181 of the *repA2* gene, responsible for plasmid replication
KPM2894	Plasmid rearrangement	IS*26* inserted at nucleotide 220 of the *repA2* gene, responsible for plasmid replication
KPM2895	Plasmid rearrangement	IS*26* inserted at nucleotide 213 of the *repA2* gene, responsible for plasmid replication, with the first 212 bp of *repA2* and upstream region missing

### Transposition of Tn*4401* into a higher-copy-number plasmid.

In mutants KPM2313 and KPM2314, selected from strain KP1084, as well as in mutant KPM2310, selected from KP1074, the 10-kb Tn*4401* transposon that contains the *bla*_KPC_ gene moved from the low-copy-number pKpQIL-like plasmid to the smaller, high-copy-number plasmid ColEST258 present in the same cell (23). In all three mutants, the transposon was identified in different locations: within the *mobC* gene, which is required for conjugation; in the Tn*3* transposase gene; and in the cloacin gene. These various locations suggest the lack of a hot spot for Tn*4401* transposition.

### Duplication of the *bla*_KPC_-containing region within the original plasmid.

Rearrangements of *bla*_KPC_-carrying plasmid pKpQIL were observed in mutants KPM2163 and KPM2164, derived from strains KP1074 and KP1084, respectively. A 22-kb region of pKpQIL (nucleotides 7046 to 29059) containing Tn*4401* and the *bla*_TEM-1_ gene was duplicated between nucleotides 2298 and 109761 in mutant KPM2163 (Fig. 1). Additionally, the 22-kb region at nucleotide 2298 and the original 22-kb region underwent homologous recombination, causing the looping out of one copy of the 22-kb region and the deletion of a 4,747-bp plasmid fragment (nucleotides 2298 to 7046) in the same mutant (Fig. 1). In KPM2164, the same 22-kb pKpQIL region (nucleotides 7046 to 29059) was duplicated *in situ*, resulting in the tandem repeat of this region. Consequently, both KPM2163 and KPM2164 carry two copies of Tn*4401*. The 4,748-bp region deleted in KPM2163 contains the FII replication origin, whose removal may have caused the observed plasmid and *bla*_KPC_ copy number increases. The resistance phenotype associated with the plasmid rearrangements described above was found to be stable for at least 10 passages under nonselective conditions.

### Inactivation of the *repA2* gene, responsible for plasmid maintenance.

We observed the insertion of IS*5* (in mutant KPM2893) or IS*26* (in mutants KPM2894 and KPM2895) in different locations of the *repA2* gene, which encodes a plasmid replication factor, in the three mutants derived from strain KP1099 (Table 4). The insertions led to *repA2* inactivation and the subsequent loss of plasmid replication and copy number control. Growth of these KPC-containing, meropenem-resistant mutants on nonselective agar plates (3 passages) cured the KPC-containing plasmid and produced meropenem-sensitive colonies. In contrast, parental strain KP1099 did not generate meropenem-sensitive progeny after multiple passages under the same nonselective conditions (data not shown). Based on these observations, we speculate that *repA2* gene inactivation undermined the plasmid replication machinery, resulting in the high frequency of loss of the KPC-carrying plasmid observed in these mutants.

### Evaluation of tigecycline resistance as a marker for increased efflux due to resistance nodulation and cell division efflux pumps.

We measured tigecycline MICs in several mutants without *ompK36* changes or increased *bla*_KPC_ gene copy numbers. Tigecycline MIC differences were not observed in mutant KPM1848, selected from strain KP1087, or in mutants KPM2804 and KPM2805, selected from strain KP1093. However, mutant KPM2327, selected from strain KP1092, displayed a 4-fold tigecycline MIC increase (from 0.5 μg/ml to 2 μg/ml; data not shown). Sequence analysis of the *ramR*, *marR*, *soxR*, and *rarR* genes, which are known to regulate efflux operon expressions in K. pneumoniae, did not identify mutations in any of these genes (20).

## DISCUSSION

In this study, 18 clinical isolates of Klebsiella pneumoniae carbapenemase (KPC)-producing K. pneumoniae were identified from large multinational meropenem-vaborbactam surveillance studies designed to understand potential resistance to this antimicrobial combination. We determined the incidence of single-step mutations that led to meropenem-vaborbactam resistance and calculated the concentrations of both agents that reduced the mutant selection frequency to a value of 10^−9^ or less. The molecular mechanisms associated with increased meropenem-vaborbactam MICs were determined for the mutants selected for further characterization.

The meropenem-vaborbactam MICs for the strains selected for this study had a broad distribution, ranging from ≤0.06 μg/ml to 32 μg/ml. In this set, 44% of the strains had M-V MICs of ≥2 μg/ml, whereas approximately 2% of KPC-producing Enterobacteriaceae strains reported in recent surveillance studies had M-V MICs of ≥2 μg/ml (13–15). The study strains were thus enriched with KPC-producing strains that had M-V MCs at the high end of the distribution of M-V MIC values and that also contained other previously described non-β-lactamase-mediated carbapenem resistance mechanisms that may be encountered in the clinic (24). The collection was phylogenetically diverse and included strains of several multilocus sequence types.

Mutant selection experiments determined that combining meropenem at 8 μg/ml with vaborbactam at 8 μg/ml was sufficient to suppress the mutant emergence frequency to 10^−9^ in 14 of 18 strains. The M-V MICs for these 14 parent strains were ≤8 μg/ml. For the remaining strains, the selection of resistant mutants was reduced by 16 μg/ml meropenem in combination with 8 μg/ml of vaborbactam. The drug concentrations used in these experiments were selected on the basis of the dosage regimens for meropenem-vaborbactam used to support controlled clinical trials (17, 18). The meropenem dose of 2 g infused over 3 h every 8 h was designed to supply concentrations above 8 mg/liter for at least 40% of a dosing interval (2). Similarly, vaborbactam concentrations of 8 mg/liter are consistent with average plasma concentrations in humans and a free 24-h area under the concentration-time curve of 192 mg · h/liter (8 mg/liter · 24 h) for a 2-g dose administered every 8 h. Phase 1 studies with both components show that these concentrations are readily obtained in humans (16, 25) and prevent resistance selection in a hollow-fiber model of infection (26).

Characterization of the mutations selected with low drug concentrations showed that the resistant phenotypes recorded in most mutants were associated with previously described carbapenem resistance mechanisms (21, 23, 27–29). These included *ompK36* inactivation due to various insertions, deletions, nonsense mutations, or substitutions and an increase in the *bla*_KPC_ gene copy number. We observed a higher tigecycline MIC in at least one mutant, suggesting an enhanced efflux activity mediated by intrinsic multidrug resistance efflux pumps, such as AcrAB-TolC. These results are consistent with the findings of a recent study with a panel of K. pneumoniae isogenic strains (30). Similar mechanisms that contribute to reduced susceptibility to ceftazidime-avibactam were recently reported (31–34). Importantly, mutations in the *bla*_KPC_ coding region were not identified in any of the mutants, including those selected from the parental strains that contained other resistance determinants, such as strains KP1096 and KP1092. In contrast, *bla*_KPC_ mutations were reported in *in vitro* selection studies of mutants with reduced susceptibility to ceftazidime-avibactam (35) and in patients treated with ceftazidime-avibactam (36).

Close examination of the laboratory-selected mutants included in this study revealed that the parental *ompK36* genetic background was an important factor that impacted the molecular mechanisms associated with the MIC increases observed in the mutants. The vast majority of mutants selected from the 5 parental strains with a fully functional OmpK36 contained various mutations in *ompK36*, and only 1 of 16 selected mutants retained the wild-type gene. Mutations in *ompK36* were associated with the highest M-V MIC value increases, up to 128-fold, with the highest M-V MIC being 8 μg/ml. In contrast, the majority of mutants (22 out of 25) originating from parental strains with a partially functional OmpK36 due to the presence of a GD duplication that constricts the porin's channel (22, 23) displayed a different mechanism, including an increase in the *bla*_KPC_ copy number, leading to up to a 32-fold increase in M-V MIC values. Thus, it appears that increasing the *bla*_KPC_ gene copy number becomes the major cause of the meropenem-vaborbactam MIC increase in the background of a partially functional OmpK36.

We sequenced the KPC-containing plasmids isolated from 8 mutants selected from 3 parents to analyze the molecular mechanisms leading to increased *bla*_KPC_ copy numbers and to elucidate if the plasticity of KPC-containing plasmids recorded in early studies could explain the observed changes (Table 4) (19, 37, 38). Three potential mechanisms that increase the *bla*_KPC_ copy number were identified: (i) intracellular transposition of the *bla*_KPC_-carrying Tn*4401* from a large, low-copy-number plasmid to a much smaller, high-copy-number plasmid; (ii) internal rearrangements of a KPC-carrying plasmid (a pKpQIL-like plasmid in our case) resulting in either an increased copy number of *bla*_KPC_ per plasmid or an increase in the number of KPC-carrying plasmids per cell; and (iii) insertional inactivation of the *repA2* gene, which controls plasmid replication (39), resulting in a loss of plasmid replication control. As a result, the plasmid copy number may be increased under selective pressure and the plasmid may be easily lost under permissive conditions.

Notably, no increase in *bla*_KPC_ copy number was detected in mutants derived from the most resistant strains, KP1096 and KP1092, which had loss-of-function mutations in both major porin genes. These mutants had the lowest increase in M-V MIC values (2- to 4-fold) of all mutant/parent groups analyzed in this study. Tigecycline MICs were elevated in one of the mutants selected from KP1092, which is consistent with increased efflux activity, a compensating mechanism that may explain the small M-V MIC increase recorded for this mutant. More studies (whole-genome sequence analysis) are under way to identify potential mutations associated with the M-V MIC increases in the remaining mutants.

In conclusion, this study identified the meropenem-vaborbactam concentrations associated with the selection or prevention of resistant mutants *in vitro*. When both meropenem and vaborbactam were used at 8 μg/ml, the selection of drug-resistant mutants was avoided in the majority of KPC-producing K. pneumoniae strains. Increasing concentrations of meropenem prevented mutant selection in all strains, including two strains with M-V MICs of 16 and 32 μg/ml. These exposures are readily obtained in plasma using the dosage regimens of meropenem and vaborbactam tested in clinical trials. The use of optimal exposures for meropenem-vaborbactam to minimize resistance emergence at infection sites is an essential strategy for the long-term clinical utility of this novel carbapenem–β-lactamase inhibitor combination.

## MATERIALS AND METHODS

### Bacterial strains.

The Klebsiella pneumoniae carbapenemase (KPC)-producing clinical isolates used in the resistance development studies were collected by JMI Laboratories (North Liberty, IA) as a part of the SENTRY Antimicrobial Surveillance Program (Table 1). KPM1275 was constructed by transferring a *bla*_KPC-3_ gene-carrying plasmid from clinical isolate KP1084 to KPM1026a, a streptomycin-resistant mutant of ATCC 43816.

### Culture media and antibiotic susceptibility testing.

Bacteria were cultured on Mueller-Hinton agar and tested on cation-adjusted Mueller-Hinton broth (CAMHB) (Becton Dickinson, Sparks, MD). Stock solutions of meropenem (Sandoz, Princeton, NJ) (10 to 50 mg/ml in distilled H_2_O) and vaborbactam (The Medicines Company, San Diego, CA) (5 to 10 mg/ml in 90% dimethyl sulfoxide) were stored at −80°C until use.

Broth microdilution susceptibility testing was performed according to Clinical and Laboratory Standards Institute methods (40), using panels prepared in-house. A checkerboard assay conforming to the Moody procedures described in the *Clinical Microbiology Procedures Handbook* (41) was used to evaluate the effect of various concentrations of vaborbactam on the meropenem MIC.

### Single-step mutant selection.

Mutants with increased meropenem-vaborbactam MICs were selected from 18 KPC-producing K. pneumoniae strains after a single-step exposure to various combinations of meropenem and vaborbactam. Approximately 5 × 10^8^ cells from fresh cultures were transferred to agar plates containing various combinations of meropenem and vaborbactam at final concentrations of between 0.5 and 16 μg/ml each. Colonies were incubated for 24 h at 37°C before the frequency of resistance emergence was calculated as the ratio of number of CFU grown on the antibiotic-containing plate over the number of CFU recorded for antibiotic-free plates. Single colonies from antibiotic-containing plates were grown twice on a nonselective medium before their antimicrobial resistance levels were assessed. Mutants with ≥4-fold increases in the meropenem MIC in the presence of vaborbactam at 8 μg/ml over the parental strain's MIC were selected for further studies.

### Gene sequencing.

The *bla*_KPC_ gene and its promoter region were PCR amplified from the majority of strains using primers KPC-promoter-F (5′-ATTCCAAACCCGAATGATCC-3′) and KPC-down-R (5′-CTCCGAATGGTTGGATCAAG-3′). Primers KPC-5451-F (5′-TGGCCAGGATGTACAACGTC-3′) and KPC-down-R were used for analysis of KPC in strain KP1099 and its mutants. Porin genes *ompK35* and *ompK36* were amplified and sequenced using primers KP-ompK35-seq-F (5′-CAGACACCAAACTCTCATCAATGG-3′) and KP-ompK35-seq-R3 (5′-AAGGGAAATCCGCTATCAGG-3′) or KP-ompK36-seq-F (5′-CAGCACAATGAATATAGCCGAC-3′) and KP-ompK36-seq-R2 (5′-TCCATTAATCGAGGCTCCTC-3′). Sequence analysis was done at Eton Biosciences (San Diego, CA).

### MLST analysis.

PCR conditions, the sequencing primers used for multilocus sequence typing (MLST), and designation of the sequence types of the K. pneumoniae isolates were based on protocol 2 of the K. pneumoniae MLST schemes available at the Pasteur Institute website (http://bigsdb.pasteur.fr/klebsiella/primers_used.html).

### *bla*_KPC_ gene copy number determination.

Single colonies grown in CAMHB with shaking at 37°C until mid-logarithmic phase were washed, boiled, and stored at −20°C until analyzed. Quantitative PCR was performed on an ABI Prism 7000 sequencing instrument (Applied Biosystems, Foster City, CA) using a SYBR detection mix (Thermo Fisher, Waltham, MA) and the primers KPC-qF2 (5′-CGCTGGTTCCGTGGTCACCC-3′) and KPC-qR2 (5′-GGCGGCGGCGTTATCACTGT-3′). Universal primers for the conserved region of the bacterial 16S rRNA gene, primers Univ-5-qF (5′-TCCTACGGGAGGCAGCAGT-3′) and Univ-5-qR (5′-GGACTACCAGGGTATCTAATCCTGTT-3′), were used as internal controls.

Threshold cycle (*C_T_*) values for the KPC gene were normalized to the 16S rRNA gene *C_T_* value for the same strain before the results were compared with the normalized *C_T_* values for the corresponding parental strain. The threshold cycle difference (Δ*C_T_*) between the mutant and the parent was used as a logarithmic power (log base 2) to calculate the relative copy number of the KPC gene in the mutant when the value for the parental strain was set equal to 1.

### Changes in the KPC-carrying plasmid. (i) Studies of Tn*4401* transposition into a smaller plasmid.

Plasmid DNA was isolated from various mutants and transformed into Escherichia coli DH5α. Transformants were selected on LB agar containing aztreonam at 2 μg/ml. The presence of the KPC gene was confirmed by PCR. The KPC-carrying plasmids were isolated from the transformants and analyzed by PCR using primers based on the published sequence of a relatively small plasmid, ColEST258 (GenBank accession no. JN247853), reported in ST-258 strains (23). Changes in PCR patterns were further analyzed by DNA sequencing.

### (ii) Investigation of changes in KPC-carrying, pKpQIL-like plasmids by targeted PCR.

Most parental strains used in this study have ST-258 or related genetic backgrounds. It has been reported that the KPC gene in ST-258 and related strains is located on a pKpQIL-like plasmid (23). To examine the difference in the pKpQIL-like plasmid between a mutant and its parent, a set of PCR primers based on the pKpQIL plasmid DNA sequence (GenBank accession no. GU595196) (38) was synthesized to cover the entire sequence of the approximately 113-kb plasmid. Changes in the PCR patterns between mutant and cognate parental strains were further analyzed by DNA sequencing.

### (iii) Investigation of plasmid changes in KP1099 by genomic sequencing.

Genomic DNA was extracted using a Qiagen DNeasy blood and tissue kit (catalog no. 69504; Qiagen). The DNA was sequenced at the Institute of Genomic Medicine, University of California, San Diego, CA, using an Illumina MiSeq V3 600 kit (catalog no. MS-102-2003; Illumina Inc.). *De novo* assembly of MiSeq data was performed using the SPAdes (version 3.5) algorithm at St. Petersburg University, St. Petersburg, Russia (42). Assembled contigs were annotated using the Prokka (version 1.11) program (Victorian Bioinformatics Consortium, Monash University, Melbourne, Australia) (43). Raw fastq sequence data for the parent and its mutant were subsequently aligned to the annotated parent assemblies using the DNAStar (version 13.0.2) program (DNAStar Inc., Madison, WI) to look for single nucleotide polymorphisms and structural sequence changes in the mutants.

### Accession number(s).

The sequences from the whole-genome shotgun projects were deposited at DDBJ/ENA/GenBank under accession numbers NMQM00000000 and NMQL00000000 for strains KP1099 and KPM2895, respectively.
